# Supplementary figures and images for: Characterization of the Microbiota Associated With 12-Week-Old Bovine Fetuses Exposed to Divergent in utero Nutrition
Source: Front Microbiol. 2022 Jan 20;12:771832. doi: 10.3389/fmicb.2021.771832 (PMC8811194; doi:10.3389/fmicb.2021.771832)

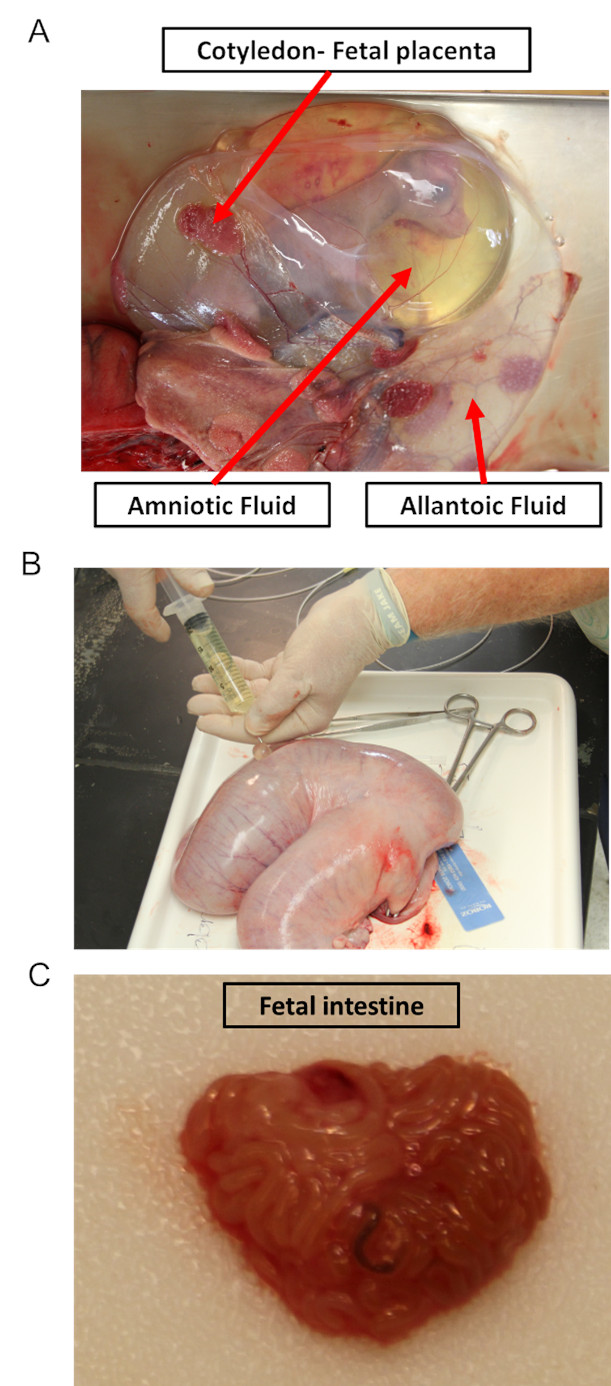

Supplement: Supplementary Figure 1 — (A) The anatomic location of amniotic and allantoic fluid, and fetal placenta (cotyledon), (B) the collection of the fetal fluids, and (C) the fetal intestine of the 83-day-old calf fetuses. [file Image_1.JPEG]

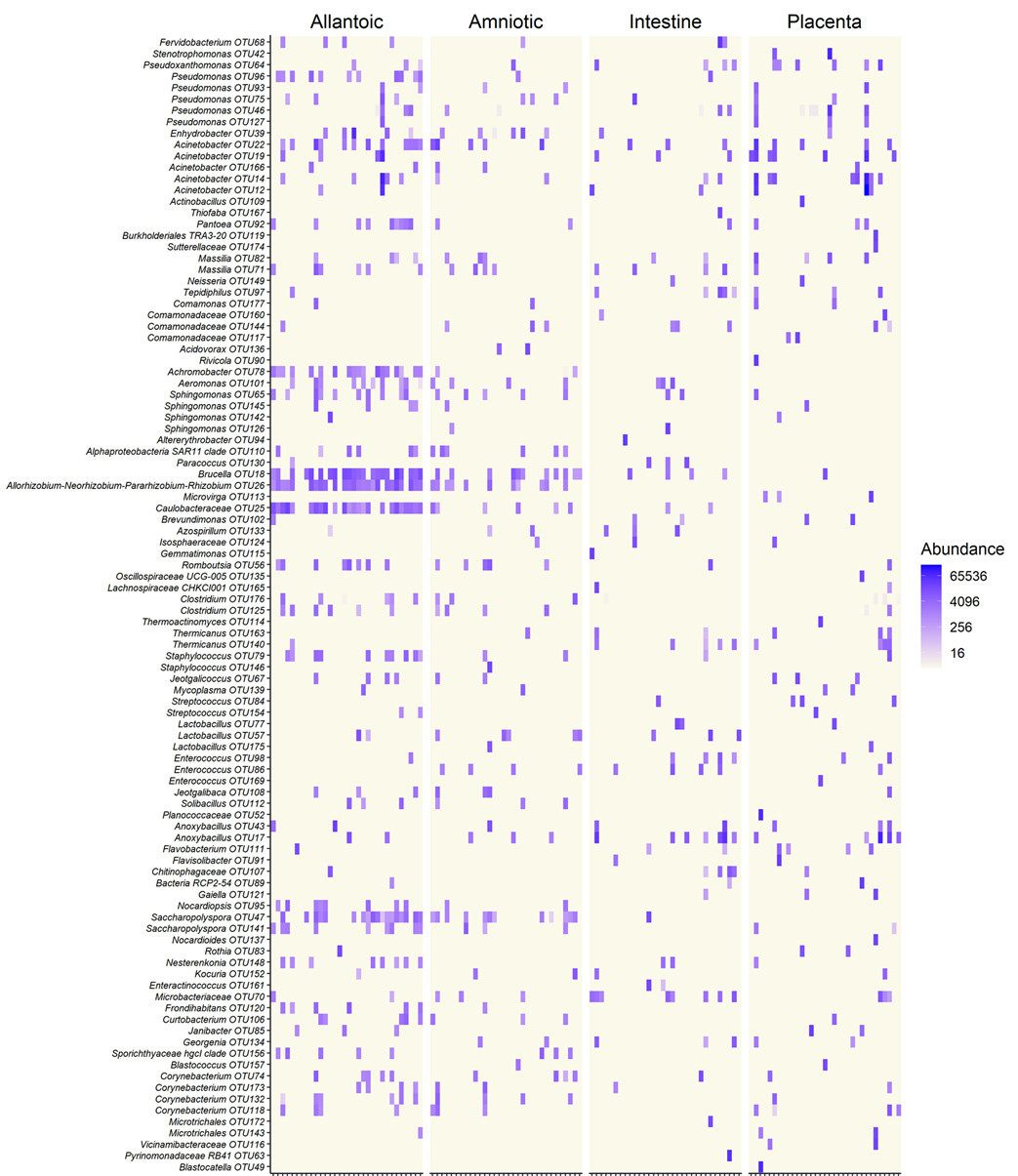

Supplement: Supplementary Figure 2 — Heatmap showing the 100 most abundant OTUs (log4) within the allantoic and amniotic fluid, and intestinal and placental microbiota in 83-day-old calf fetuses. [file Image_2.JPEG]
