# Supplementary material for: Characterization of the Microbiota Associated With 12-Week-Old Bovine Fetuses Exposed to Divergent in utero Nutrition
Source: Front Microbiol. 2022 Jan 20;12:771832. doi: 10.3389/fmicb.2021.771832 (PMC8811194; doi:10.3389/fmicb.2021.771832)
Supplement: Supplementary file 3 [file Data_Sheet_1.PDF]

Supplementary Table S1. Overall relative abundance (%) of archaeal families present in allantoic and amniotic fluid, and intestinal and placental tissue samples obtained from 83-day-old calf fetuses.

**Archaeal family**	**Allantoic**	**Amniotic**	**Intestine**	**Placenta**
k__Archaea;p__Crenarchaeota	0.094	0.000	0.204	0.611
k__Archaea;p__Euryarchaeota	0.018	0.093	0.152	0.025
k__Archaea;p__Nanoarchaeota	0.000	0.152	0.000	0.000
k__Archaea;p__Crenarchaeota;c__Nitrososphaeria	0.094	0.000	0.204	0.611
k__Archaea;p__Euryarchaeota;c__Methanobacteria	0.018	0.093	0.152	0.025
k__Archaea;p__Nanoarchaeota;c__Nanoarchaeia	0.000	0.152	0.000	0.000
k__Archaea;p__Crenarchaeota;c__Nitrososphaeria;o__Nitrosopumilales	0.094	0.000	0.000	0.000
k__Archaea;p__Crenarchaeota;c__Nitrososphaeria;o__Nitrososphaerales	0.000	0.000	0.204	0.611
k__Archaea;p__Euryarchaeota;c__Methanobacteria;o__Methanobacteriales	0.018	0.093	0.152	0.025
k__Archaea;p__Nanoarchaeota;c__Nanoarchaeia;o__Woesearchaeales	0.000	0.152	0.000	0.000
k__Archaea;p__Crenarchaeota;c__Nitrososphaeria;o__Nitrosopumilales;f__Nitrosopumilaceae	0.094	0.000	0.000	0.000
k__Archaea;p__Crenarchaeota;c__Nitrososphaeria;o__Nitrososphaerales;f__Nitrososphaeraceae	0.000	0.000	0.204	0.611
k__Archaea;p__Euryarchaeota;c__Methanobacteria;o__Methanobacteriales;f__Methanobacteriaceae	0.018	0.093	0.152	0.025
k__Archaea;p__Nanoarchaeota;c__Nanoarchaeia;o__Woesearchaeales;f__NA	0.000	0.152	0.000	0.000

Supplementary Table S2. *P-values* for the comparison in alpha diversity metrics and relative abundance of the five relatively most abundant phyla associated with different fetal samples between treatment groups.

P-values	**Allantoic**			**Amniotic**			**Intestine**			**Placanta**		
	Gain	VTM	Gain × VTM	Gain	VTM	Gain × VTM	Gain	VTM	Gain × VTM	Gain	VTM	Gain × VTM
Alpha diversity												
Number of OTUs	0.055	0.295	0.459	0.828	0.880	0.359	0.477	0.226	0.054	0.366	0.765	0.241
Shannon diversity index	0.302	0.311	0.565	0.654	0.772	0.122	0.723	0.805	0.100	0.626	0.942	0.740
Inverse Simpson's index	0.453	0.536	0.191	0.862	0.851	0.180	0.828	0.927	0.117	0.454	0.935	0.463
Top 5 phyla												
Proteobacteria	0.920	0.872	0.743	0.188	0.448	0.040	0.163	0.002	0.000	0.755	0.610	0.630
Actinobacteriota	0.561	0.691	0.786	0.626	0.395	0.119	0.721	0.905	0.668	0.461	0.789	0.763
Firmicutes	0.760	0.735	0.149	0.640	0.732	0.919	0.645	0.006	0.343	0.168	0.066	0.692
Bacteroidota	0.760	0.735	0.149	0.057	0.363	0.173	0.554	0.741	0.308	0.934	0.198	0.806
Cyanobacteria	0.687	0.696	0.341	0.169	0.014	0.262	NA	NA	NA	NA	NA	NA
Acidobacteriota	NA	NA	NA	NA	NA	NA	0.125	0.766	0.123	0.241	0.636	0.082
